# Measuring nanoparticles in liquid with attogram resolution using a microfabricated glass suspended microchannel resonator

**DOI:** 10.1038/s41378-022-00425-8

**Published:** 2022-08-30

**Authors:** Mehdi Mollaie Daryani, Tomás Manzaneque, Jia Wei, Murali Krishna Ghatkesar

**Affiliations:** 1 Department of Precision and Microsystems Engineering, Delft University of Technology, Delft, The Netherlands; 2 Department of Microelectronics, Delft University of Technology, Delft, The Netherlands; 3 Present Address: Department of Microelectronics, Delft University of Technology, Delft, The Netherlands

**Keywords:** Nanofluidics, Nanosensors

## Abstract

The use of nanoparticles has been growing in various industrial fields, and concerns about their effects on health and the environment have been increasing. Hence, characterization techniques for nanoparticles are essential. Here, we present a silicon dioxide microfabricated suspended microchannel resonator (SMR) to measure the mass and concentration of nanoparticles in a liquid as they flow. We measured the mass detection limits of the device using laser Doppler vibrometry. This limit reached a minimum of 377 ag that correspond to a 34 nm diameter gold nanoparticle or a 243 nm diameter polystyrene particle, when sampled every 30 ms. We compared the fundamental limits of the measured data with an ideal noiseless measurement of the SMR. Finally, we measured the buoyant mass of gold nanoparticles in real-time as they flowed through the SMR.

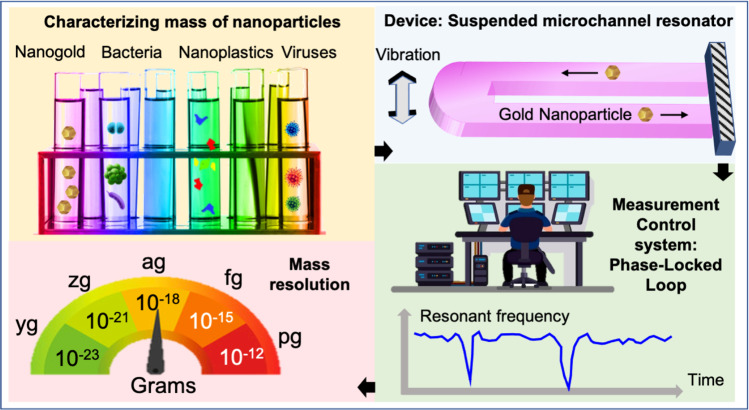

## Introduction

Plastic products are used in many fields from health to textiles and construction. The world plastic production has increased dramatically, from 2 million tons in 1950 to almost 368 million tons in 2019^1,2^. Synthetic polymers such as polyethylene (PE), polystyrene (PS), polyurethane (PUR), polypropylene (PP) and polyvinyl chloride (PVC) are the core of plastics. In addition to polymers, additives are added to plastics to enhance their mechanical, chemical and visual characteristics such as strength, fire resistance, and coloration^1^. Although the potential toxicity of nanoplastics on humans is yet to be verified, it is known that nanoplastics can pass biological barriers to penetrate organs and can move across immune cells^3–5^. This shows the need for tools to assess physical and chemical properties of plastic nanoparticles. On the other hand, the development of tools for the precise identification, quantification and characterization of nanoparticles suspended in liquid has been limited. These nanoparticles include not only nanoplastics but also exosomes, viruses, protein aggregates, and so on^6,7^. Most available analysis approaches are based on light scattering, such as multiangle light scattering (MALS) and dynamic light scattering (DLS). A major limitation of these methods is that they are prone to errors in polydisperse fluids^8,9^. Hence, there still is a demand for precise characterization methods to assess nanoparticles irrespective of their composition and heterogeneity..

Suspended microchannel resonators have emerged as a powerful characterization tool for micro- and nano-scale particles^10^. These devices allow for measuring the buoyant mass and concentration of particles suspended in liquid in a flow-through mode^7,11,12^. The buoyant mass is defined as *m*_b_ = *V* × (*ρ*_o_ − *ρ*_l_), where *V* is the volume of the object, *ρ*_o_ is the density of the object and *ρ*_l_ is the density of the liquid in which the object is immersed. The possibility of operating the resonator in vacuum and subsequent high quality factors enable mass resolutions of attograms for particles in liquid^12^, not accessible by traditional resonators working under full immersion.

Here, we report on the limits of detection or mass resolution of silicon dioxide suspended microchannel resonant cantilevers. An analysis of the factors limiting the resolution of the device is presented, and evidence of nanoparticles detection is shown.

## Methods

### Suspended microchannel resonator (SMR) device

**Fig. 1:**
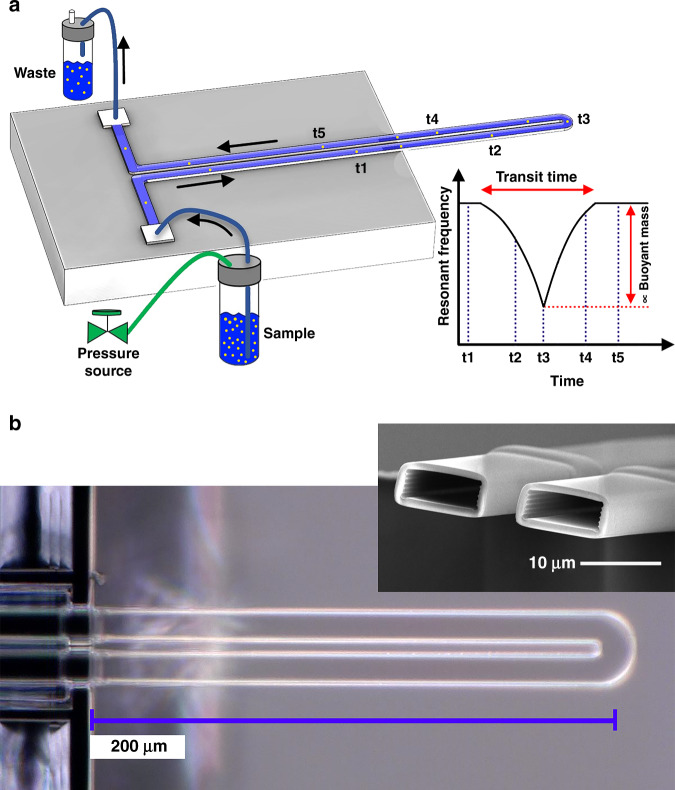
Experimental schematic and device description. **a** Schematic of the experimental setup to determine the buoyant mass of nanoparticles using a cantilever-shaped suspended microchannel resonator (SMR). Particles flow through the channel from the sample reservoir (inlet) to the waste reservoir (outlet). The time points t1 to t5 represent the position of the nanoparticle as it flows through the channel. The plot shows a representative resonant frequency shift with time for the fundamental resonant mode. **b** Optical microscope top-view image of the U-shaped silicon dioxide SMR. A scanning electrode microscope image of the cantilever cross-section is shown in the inset

Figure 1a shows a schematic picture of the microfabricated SMR chip and the experimental setup. Figure 1b shows top-view and cross-sectional images of the SMR cantilever taken by optical microscopy and scanning electron microscopy, respectively. The nanoparticle-carrying flow is injected into the microchannel at the flow inlet on-chip and collected from the flow outlet on-chip. A pressure controller establishes a continuous flow. The resonant frequency of the cantilever changes as a nanoparticle flows through the SMR. The shift in the resonant frequency is proportional to the buoyant mass of the particle and its position along the cantilever. As a result, a transient shift is obtained for a single particle, whose maximum value (minimum resonant frequency) is proportional to the nanoparticle’s buoyant mass (Fig. 1a). A SMR device is modeled as a mass-spring-damping linear system. Equation (1) gives the frequency shift obtained due to the addition of a particle buoyant mass *m*_b_ at the cantilever tip^13^. A positive buoyant mass produces a negative resonant shift Δ*f*_r_.

1 $${{\Delta }}{f}_{{\rm{r}}}=-\frac{{f}_{{\rm{r}}}}{2{m}_{{\rm{eff}}}}{m}_{{\rm{b}}},$$

where *f*_r_ and *m*_eff_ are the resonant frequency and effective modal mass of any vibration mode of the cantilever, respectively. The density and volume of the particle can be determined by Archimedes’ principle if carrying liquids of different densities are used for the same particle^14^.

### Device description

**Fig. 2:**
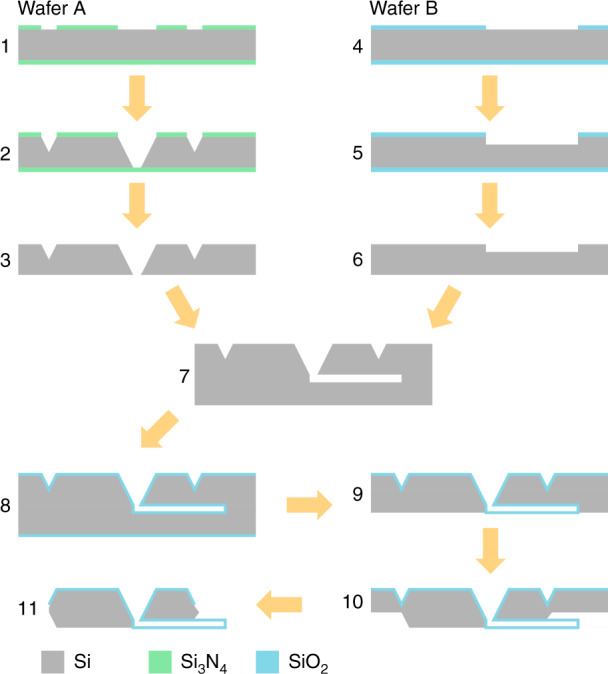
Flow chart of the fabrication process with the following steps. (1) Deposition of silicon nitride (Si_3_N_4_) by low-pressure chemical vapor deposition (LPCVD) followed by patterning by reactive ion etching (RIE). (2) Wet etching of silicon (Si) by potassium hydroxide (KOH) to form the fluid reservoirs and release trenches. (3) Removal of Si_3_N_4_. (4) Thermal oxidation of Si and patterning of the formed silicon dioxide (SiO_2_) layer by RIE. (5) Etching of Si by RIE to form the microfluidic channel cavity. (6) Removal of SiO_2_. (7) Wafer bonding. (8) Thermal oxidation of the internal wall of the microfluidic channel cavity. (9) Bottom side etching of Si by RIE. (10) Release of the SiO_2_ cantilevers by etching Si in KOH. (11) Manual release of the chips from the wafer by the trenches. The channel widths were kept below 40 μm to avoid cracks in the suspended SiO_2_ regions. At least 1.7 μm thick SiO_2_ was used to avoid cracks in the corners where the channel bends^25^

The device can be described as a silicon dioxide U-shaped suspended microfluidic channel (Fig. 1b). For the fabrication (Fig. 2), two silicon wafers were bonded together, with the microfluidic channel etched in one wafer and the flow inlet and outlet reservoirs in the other. Subsequent oxidation and KOH etching steps define the complete device. Further details of the fabrication process can be found elsewhere^15,16^. The two reservoirs on the backside of the chip connect the channel to the outside world. Table 1 shows the relevant dimensions of the device. The devices were used as fabricated without any functionalization of the channels. To facilitate and control the flow of liquids through the SMR from the outside, the chip was glued to a flow interface. The flow interface was produced using stereolithography 3D printing (Envisiontec Micro Plus, USA) with 30 μm resolution and high-temperature mold material (HTM140). The interface was ultrasonically cleaned in 99% isopropanol, followed by 2 min curing with UV light. The SMR chip was glued to the interface using epoxy adhesive (EA 9492). Prior to gluing the chip on the interface, a cleaning step was performed to avoid channel clogging by debris from the polymeric interface. This procedure was implemented in the following sequence: (a) Multiple injections of ethanol using a syringe. (b) Ultrasonic cleaning bath for 5 min. (c) Repeat step one using DI water instead of ethanol. (d) Repeat step one using isopropanol instead of ethanol. (e) Drying the interface using a nitrogen gun. Two dispensing tips (Nordson 32GA) were glued to the interface for further connection using tygon tubing with a 0.20 mm inner diameter and a 2.00 mm outer diameter.

**Table 1:** Dimensions of the device

Parameter	Value (μm)
Cantilever length	200
Channel length (suspended)	408
Gap between the legs (outer)	5.4
Channel width (outer)	11.3
Channel height (outer)	3.5
Channel wall thickness	1.7

### Test solutions

Deionized (DI) water was used for the initial characterization of the cantilever. The DI water was produced by a purifier Purelab Flex, ELGA, UK, system with a resistivity of 18.2 MΩ cm. A a 0.2 μm syringe filter was used twice to clean the water from possible contaminating particles. A stock solution of 0.05 mg mL^−1^ of 100 nm gold nanoparticles in aqueous suspension was obtained from nano-Composix. For the experiments, using a dilution factor of 1: 10^5^, a 5 mL solution volume with a concentration of 1.022 × 10^7^ particles mL^−1^ was prepared. This concentration was one order of magnitude lower than the concentration equivalent to one particle per volume of the cantilever. By this choice, the probability of having two or more particles simultaneously in the suspended channel is small.

### Measurement setup

**Fig. 3:**
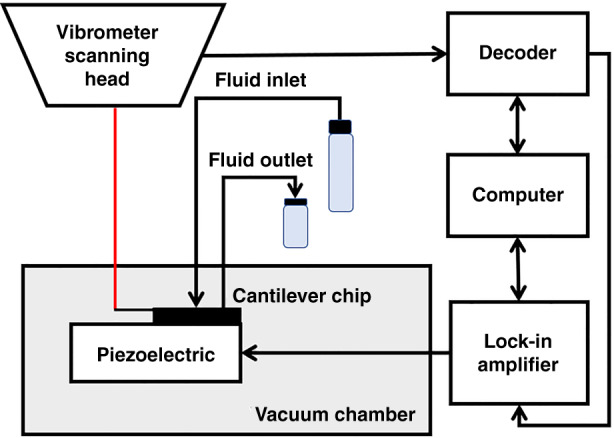
Schematic of the measurement setup. In phase-locked loop mode, the lock-in's internal oscillator was used to excite the piezo, resulting in SMR vibration. The vibrometer decoder translates the optical signal received by the scanning head into an electrical signal proportional to the cantilever velocity. A personal computer (PC) was used to control and read the data from the lock-in amplifier and the vibrometer

Experiments were performed to investigate the frequency stability of the presented device, and measure the buoyant mass of gold nanoparticles. A schematic of the experimental setup is shown in Fig. 3. The resonator was mounted on a Nanosurf AG company’s atomic force microscopy (AFM) holder (BT06062) with a piezo-actuator. The AFM holder was placed inside a vacuum chamber with an electrical feed-through for the excitation signal and flow feedthroughs for the inlet and outlet tubing. A laser Doppler vibrometer (Polytec MSA-400-PM2-D) was used for optical measurement of the resonator’s mechanical vibrations, and a digital lock-in amplifier (Zurich UHFLI) was employed to implement a frequency tracking scheme based on a phase-locked loop (PLL)^11^. The lock-in provided a sinusoidal excitation signal of 1.5 V that allowed the detection of the resonant frequency below the onset of nonlinear effects. To avoid saturating the input of the lock-in, a 10 dB attenuator was used between the vibrometer output and the lock-in input. To set the correct PLL configuration, initially, several frequency sweeps with increasing actuation voltage values were performed around the target resonant frequency, until nonlinear effects were visible by the deformation of the resonance curve. The highest voltage below the onset of non-linearity was set for the remainder of the experiment. Next, the required parameters for setting a PLL control scheme, namely quality factor (*Q*), resonant frequency and phase value at resonance, were determined. Then, the PLL was implemented based on the theory described by Olcum et al.^11^. Ultimately, by enabling the PLL, the response of the resonator’s phase and frequency were measured over a period of 5 min.

### Device characterization

To characterize the resonator in terms of stiffness (*k*), effective mass (*m*_eff_) and quality factor (*Q*) in both empty and filled cases, the following steps were taken. First, a frequency spectrum was measured while exciting the cantilever vibrations around the resonance of interest. The quality factor was determined from this data using the 3dB method. Then, the cantilever vibrations were recorded in the same frequency range without piezoelectric actuation. The recorded vibrations were translated into the frequency domain by fast Fourier transforms (FFT) and averaged over 500 measurements. The maximum power spectral density (PSD) value, obtained at the resonant frequency, allowed for calculating the effective stiffness *k* and mass *m*_eff_ of the vibration mode^17^:

2 $$k=\frac{4{K}_{{\rm{B}}}TQ}{{\omega }_{{\rm{r}}}PSD({\omega }_{{\rm{r}}})},$$

3 $${m}_{{\rm{eff}}}=\frac{k}{{\omega }_{{\rm{r}}}^{2}},$$

where *T* is the absolute temperature, *K*_B_ is Boltzmann’s constant, *ω*_r_ is the resonant frequency and PSD(*ω*_r_) is the power spectral density of the resonator’s displacement evaluated at the resonant frequency.

### Data processing

To investigate the frequency stability, the Allan deviation (*σ*_*y*_) was computed as shown in Eq. (4). The frequency data were recorded by the PLL setup, as a function of the gate time (*τ*) for the (*M*) number of frequency samples^18^:

4 $${\sigma }_{y}(\tau )=\sqrt{\frac{1}{M}\mathop{\sum }\limits_{k=0}^{M-1}\frac{{({\overline{y}}_{k+1}-{\overline{y}}_{k})}^{2}}{2}},$$

where $\overline{y}$ is the averaged fractional frequency over a gate time period.

The theoretical Allan deviation due to thermomechanical noise was also calculated using the following expression^19^:

5 $${\sigma }_{y}(\tau )=\sqrt{\frac{{m}_{{\rm{eff}}}{\omega }_{{\rm{r}}}{k}_{{\rm{B}}}T}{{{A}_{0}}^{2}{Q}^{3}\tau }},$$

where *A*_0_ is the applied force at resonance and *m*_eff_ is the effective mass found by Eq. (3). The applied force can be determined from the measured displacement amplitude at resonance (*X*_0_) as

6 $${A}_{0}=\frac{{X}_{0}{k}_{{\rm{eff}}}}{Q}.$$

The measured and theoretical Allan deviations obtained by Eqs. (4) and (5) can be related to the limit of mass detection as follows^20^:

7 $$\delta m\approx -2{m}_{{\rm{eff}}}{\sigma }_{y}(\tau ).$$

## Results and discussion

### Suspended microchannel resonator characterization

**Fig. 4:**
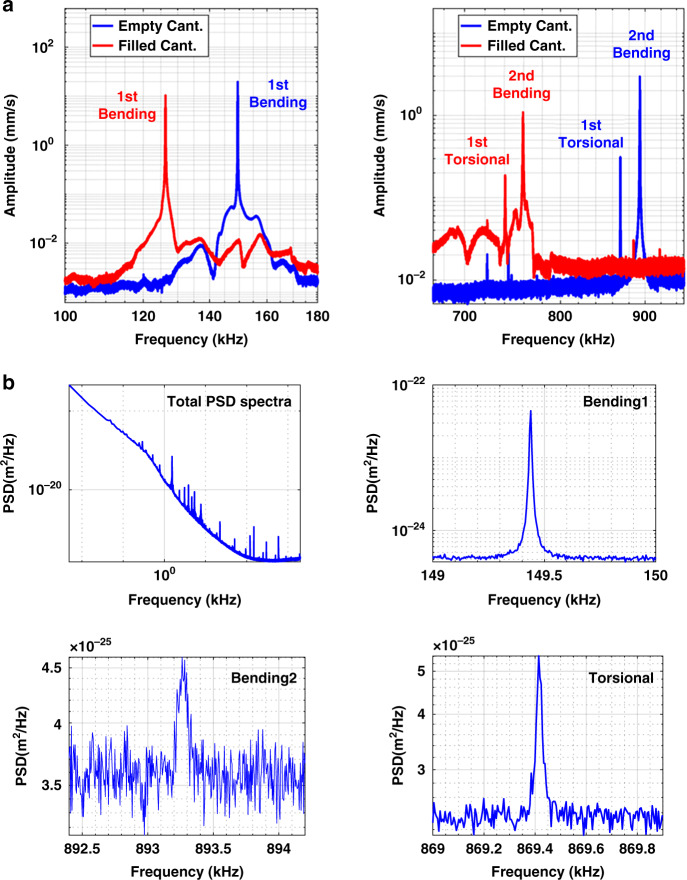
Measured frequency spectra and power spectral density of the resonator. **a** Complete spectra of the empty and water-filled resonator up to 2 MHz. **b** PSDs of the first two bending modes and first torsional mode with no excitation signal applied, used for the determination of the effective stiffness and mass of each vibration mode

A modal analysis of the device was performed by optical Doppler vibrometry to obtain the resonant frequencies and quality factors of different modes of the cantilever over a bandwidth of 2 MHz. The results are shown in Fig. 4a. The power spectral density data shown in Fig. 4b, obtained without cantilever excitation, were used to obtain the stiffness and effective mass of each mode by applying Eqs. (2) and (3). These parameters were measured both when the resonator was empty and filled with water. In the case of the water-filled cantilever, DI water was pumped by applying a pressure difference of 70 mbar between the inlet and outlet reservoirs. It was noticed that the fill time showed variations between 1 s and 25 s for the same applied pressure over nominally identical devices. This indicates a large variation in the hydrodynamic resistance of the devices. This could be due to partial blockage of the inlet or/and outlet flow reservoirs due to the gluing of the chip, or due to residues in channels during SMR fabrication.

In a frequency range up to 2 MHz, three modes were identified in a vacuum environment of 0.012 mbar. All modes were present at frequencies below 1 MHz, including two bending modes and one torsional mode (see Fig. 4a). The important parameters of the detected modes are shown in Table 2. There might be higher modes between 1 and 2 MHz; however, due to their low amplitudes, they could be hidden under the noise background of the measured spectra. The full range spectra up to 2 MHz can be seen in the supplementary information. In the frequency spectra, several other spikes were also observed. These peaks, which do not correspond to modal shapes of the cantilever, are rigid body motions of the chip or its base, a result of using base excitation by an external piezoelectric element. The water-filled resonator experienced reductions in the resonant frequencies of 23 kHz, 134 kHz, and 129 kHz for first bending, second bending, and torsional modes, respectively. These shifts in resonant frequencies are due to the increase in the effective mass of the water-filled cantilever, which is inversely proportional to the resonant frequency. The predicted values from COMSOL simulation (see supplementary notes) of the resonant frequencies (112.24 kHz, 674.45 kHz, and 698.54 kHz) are lower than those obtained experimentally for the empty cantilever. This discrepancy is attributed to a possible variation in the thickness of the channel, which might be slightly non-uniform as a result of the fabrication process.

**Table 2:** Summary of the resonator’s parameters for the different vibration modes analyzed, obtained from the modal characterization experiment

Channel condition	Mode	Resonant frequency (kHz)	Quality factor	Effective mass (ng)	Allan deviation (*σ*_*y*_)	Mass limit (ag)	AuNP (nm)	PSNP (nm)
Empty	1^st^ Bending	149.450	25993	81.1	1.62 × 10^−9^	263	30	216
	2^nd^ Bending	893.287	13214	17.1	3.09 × 10^−9^	106	22	159
	1^st^ Torsional	869.428	43634	50.4	1.48 × 10^−8^	1.49 × 10^3^	54	384
Filled	1^st^ Bending	126.393	9716	113	7.82 × 10^−9^	1.77 × 10^3^	57	407
	2^nd^ Bending	759.183	3241	23.7	7.96 × 10^−9^	377	34	243
	1^st^ Torsional	740.550	4784	69.4	1.30 × 10^−7^	18.03 × 10^3^	123	883

Minimum Allan deviation and corresponding mass detection limit, along with the smallest detectable spherical particle’s diameter for gold and polystyrene nanoparticles (Au NPs and PS NPs, respectively).

Regarding the quality factors in the empty state, the torsional mode showed the highest value and the second bending mode showed the lowest value. A reduction in the quality factor from the first to second order in bending modes is in accordance with previous work on this type of device^21^. Moreover, significant drops in the quality factor (62.6%, 75.5%, and 89% in the first bending, the second bending, and the first torsional modes, respectively) were noticed when the device was filled with water. As expected, compared to the empty case, the effective mass increased for each mode when the cantilever was filled with water.

### Frequency stability and mass resolution

A PLL bandwidth (*f*_PLL_) of 1 kHz was used. For this purpose, a low-pass filter of 4th order with a bandwidth (*f*_LPF_) of 1.41 kHz was used for phase detection. The proportional and integral controller constants were set to *K*_p_ = 4442.9 rad s^−1^ and *K*_i_ = 80233.7 rad s^−2^, respectively. A sampling frequency (*f*_s_) of 3.598 kHz was selected to collect the PLL frequency as a function of time.

**Fig. 5:**
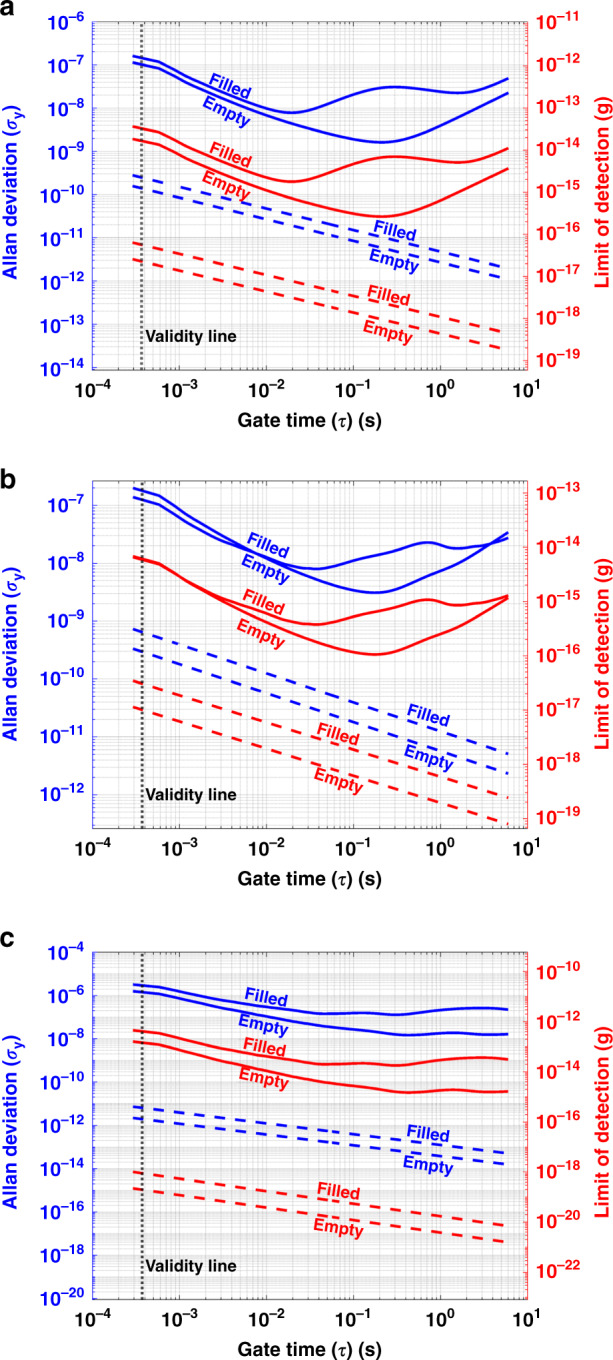
Mass sensitivty of the resonator for different vibration modes. Allan deviation and corresponding lowest buoyant mass detection limit for **a** the first bending mode, **b** the second bending mode and **c** the first torsional mode. The validity line (black-dotted line) is determined by (2.33/*ω*_PLL_). The blue-dashed and red-dashed lines represent the Allan deviation that can be achieved theoretically by our system if only thermomechanical noise is present

The frequency stability (left vertical axis) and mass detection limit (right vertical axis) of the resonator when empty and filled with water are plotted in Fig. 5, for the already mentioned PLL parameters. In all figures, the validity line shown in vertical black dots specifies the minimum gate time that the PLL is able to resolve. This line delimits the gate time range for which the measured Allan deviation truly represents the stability of the measured resonant frequency, *τ* > 2.33/*ω*_PLL_^22^. For shorter gate times, the PLL transfer function introduces a significant filtering of the system noise, which artificially reduces the Allan deviation and thus the apparent mass detection limit. Therefore, the validity line specifies the maximum detection speed of the implemented system. This speed is 0.37 ms for a 1 kHz PLL bandwidth.

The measured Allan deviations for the empty and water-filled conditions of the resonator, in all detected modes, demonstrate similar trends. It is observed that there is a continuous reduction in *σ*_*y*_ for growing gate times below certain value, between 20 ms and 400 ms, where the minima for all modes are reached for the empty and water-filled measurements respectively. In both cases, the Allan deviations at the minima have almost the same magnitude for the first and second bending modes. These minima values are approximately one order of magnitude larger (noise level is higher) in the case of the first torsional mode compared to the first and second bending modes. For gate times longer than that of the minima, the curves follow a positive slope in the case of the first and second bending modes, while almost a zero slope for the torsional mode. The zero slope in the Allan deviation is an indication of 1/*f* frequency noise (flicker), see ref. ^18^, while the positive slope at long gate times is attributed to the thermal drift due to slow variations in temperature.

The Allan deviation increases when the cantilever is filled with water. This increase is especially pronounced for the first and second bending modes for gate times between 0.02 ms and 3 s. This phenomenon can be attributed to temperature fluctuations induced by the measurement laser on the cantilever. In principle, we could expect a reduction in the Allan deviation when the liquid is present inside the cantilever, due to the mitigation of these laser-induced temperature fluctuations with the increased thermal conductivity. Furthermore, the presence of the liquid also brings a reduction in the thermal time constant of the device^23^. Thus, laser-induced variations in the resonant frequency can be too slow to be seen in the measured Allan deviation when the cantilever is empty. Then, when the cantilever is filled, these variations can become smaller in magnitude but faster, now falling in the range between 0.02 ms and 3 s, where they become dominant as the Allan deviation due to other causes is minimal. This explanation is in accordance with the theory presented in Snell et al.^24^, which models temperature fluctuations as having a low-pass characteristic with thermal constant *τ*_th_. The frequency noise induced by the temperature fluctuations will present the same low-pass characteristic, which translates into an Allan deviation that grows with +1 slope for *τ* < *τ*_th_ and rolls off with −1/2 slope for *τ* > *τ*_th_^18^, in agreement with the bump in the Allan deviation seen for the filled cantilever in Fig. 5a.

Another important consideration is the fundamental limit for the Allan deviation of the device. This corresponds to the Allan deviation that would be measured in the absence of any parameter fluctuation or noise source, other than the thermomechanical noise of the device^19^. Thermomechanical noise is defined as the random vibrations of a mechanical device due to thermal motion of atoms. This noise sets the fundamental noise limit of the device, as it cannot be mitigated by controlling the experimental conditions or improving the measurement system. This limit, calculated with Eq. (5), is plotted in Fig. 5a–c for the different vibration modes analyzed (blue dash lines). From Fig. 5, we notice that there are almost two orders of magnitude of discrepancy between the thermomechanical Allan deviation and the corresponding experimental curves in the first and second bending modes. This discrepancy becomes more conspicuous for the torsional mode, where there is a difference of five orders of magnitude. These discrepancies can be explained by measurement noise introduced by the vibrometer or the lock-in amplifier that dominates the measured Allan deviation at low gate times, for both the empty and water-filled cases.

To address the minimum detectable buoyant mass, the Allan deviation curves were translated into the mass limit of detection using Eq. (7). The results are shown in Fig. 5 for the different modes in red, with the vertical axis on the right side. The smallest detectable mass by each mode, and the equivalent gold and polystyrene particle’s diameter are given in Table 2. The lowest mass detection limit is achieved by the second bending mode. Note that the minimum for the mass detection limit is found at shorter gate times when the cantilever is filled with water (20–40 ms) than when the cantilever is empty (200–400 ms).

### Particle detection

**Fig. 6:**
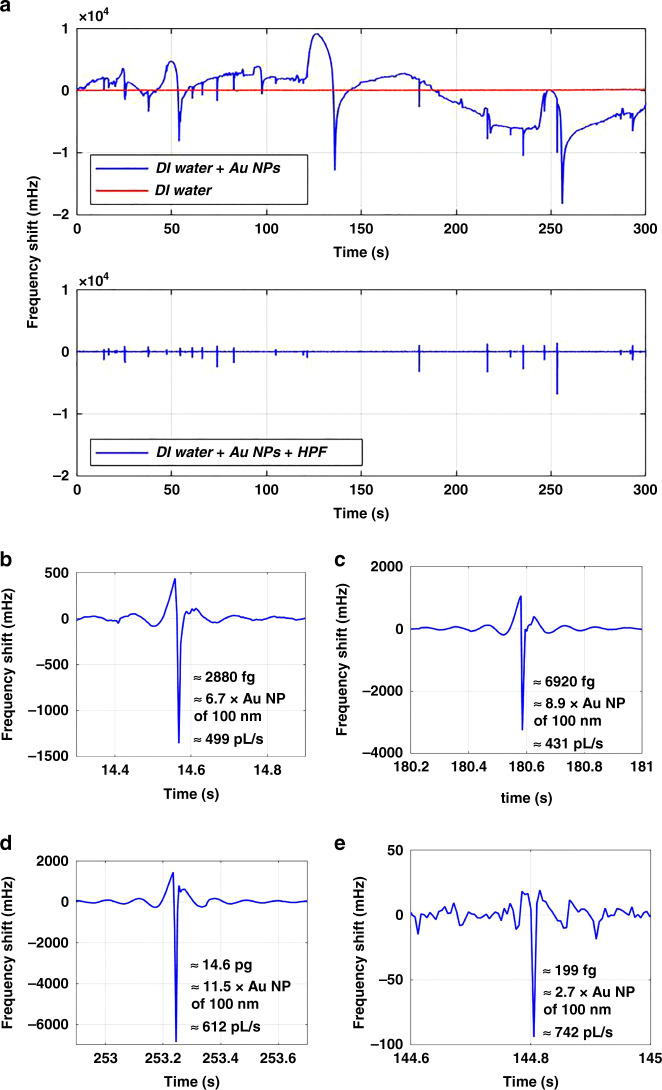
Real-time mass detection of particles as they flow through the channel. **a** (Upper panel) Comparison of frequency shifts when DI water was flowing with an applied pressure difference of 70 mbar (red), and when a solution of 100 nm diameter gold nanparticles was flowing with an applied pressure difference of 500 mbar (blue). (Lower panel) The data obtained after applying a high-pass filter with a cut-off frequency of 10 Hz on the data with nanoparticles flowing through the channel (blue curve in the upper panel). **b**–**e** Zoom-in views from **a** corresponding to particle detection events. The calculated masses and their equivalent Au particle’s diameters are shown, along with the flow rates, estimated from the particle transient time

To demonstrate the detection of particles by our resonator, a liquid solution containing gold nanoparticles was injected into the channel and the first bending mode was monitored. The results for an applied pressure difference of 500 mbar are shown in Fig. 6. The result for a pressure difference of 1200 mbar is shown in the supplementary material.

Using the described PLL system, the resonant frequency was recorded for a period of 300 s. To target the limit of detection of the first bending mode, the obtained data points were averaged over periods of 5 ms. The PLL was set with a 4th order low-pass filter of 100 Hz bandwidth, *K*_p_ = 444.3 rad s^−1^ and *K*_*i*_ = 8023.4 rad s^−2^, resulting in a PLL settling time of 3.7 ms, which is faster than the selected averaging time (5 ms) for processing the results. The evolution of the resonant frequency with time can be seen in Fig. 6a for the case of the gold nanoparticle solution, along with the case of only DI water flowing through the resonator. Frequency shifts are observed for the gold nanoparticle solution which are not present for the experiment with DI water. The blips in the frequency shift are due to the flow of particles or aggregates of particles through the cantilever. In addition to the small frequency shifts as blips, there are also much larger frequency variations that are repeated regularly over time. These drastic variations start with a considerable rise to reach a maximum, then followed by a rapid plunge to a minimum (see an example of them in Fig. 6a, from 100 to 150 s). It was noted that they were repeated more frequently when the applied pressure difference across the microfluidic channel was increased (see supplementary notes). A possible explanation for the large shifts is the accumulation of particles at places where the channel gets partially clogged, building up the channel pressure. When the pressure is high enough to release the blockage, a sudden decrease in pressure is produced, that finally stabilizes to the original pressure bringing the resonant frequency back to the baseline.

For the observed frequency shifts, we attribute fast changes to nanoparticles and slow changes to the pressure fluctuations as explained above. To remove the latter, we applied a finite impulse response (FIR) high-pass filter of 10 Hz cut-off frequency to the data. A comparison between the filtered and unfiltered data is shown in Fig. 6a. As soon as the unwanted frequency variations were filtered, the smooth frequency shifts over time were monitored to find distinguished peak profiles. Some examples of these variations attributed to flowing nanoparticles are shown in Fig. 6b–e, together with their calculated masses. Notably, the smallest found frequency shift was −10 mHz, which is equivalent to a gold nanoparticle of 130 nm (buoyant mass of 21.2 fg). This is in good agreement with the nominal diameter of 100 nm reported by the manufacturer.

## Conclusions

In this work, we have characterized the frequency stability and mass detection limits of silicon dioxide suspended microchannel resonators. In terms of frequency stability, for short gate times (*τ* < 20 ms when the channel was filled with water and *τ* < 200 ms in the empty case), the measured stability was limited by instrumentation noise in all three modes analyzed. However, for long gate times, thermal drift and flicker noise were dominant in the bending modes and the torsional mode, respectively.

In terms of mass detection limit, the best mass resolution was obtained by the second bending mode, followed by the first bending mode and the torsional mode. For a PLL bandwidth of 1 kHz, the lowest resolved mass for an empty and a water-filled resonator using the second bending mode was 106 ag and 377 ag, respectively, which corresponds to a system settling time of 0.37 ms.

The ability of the system to detect gold nanoparticles suspended in water was demonstrated, with a measured mass of 21.2 fg, which is equivalent to a 130 nm diameter gold nanoparticle. The novelty of the work lies in the device being like atomic force microscopy (AFM) cantilever but without a pyramid tip, which is capable of detecting attograms of buoyant mass. With few additional steps in the fabrication process, a fluid dispensing/aspirating AFM tip can be fabricated.

Based on these results, our sensor is capable of measuring the mass of nanoparticles continuously, precisely and with high throughput. The next steps will be to push the noise floor in the measurements further down to reach the thermomechanical noise limit, eliminate the undesired pressure pulses in the flow, and vacuum package the device for practical applications.

## Supplementary information

Measuring nanoparticles in liquid with attograms resolution using microfabricated glass suspended microchannel resonator
