# Supplementary material for: Measuring nanoparticles in liquid with attogram resolution using a microfabricated glass suspended microchannel resonator
Source: Microsyst Nanoeng. 2022 Aug 30;8:92. doi: 10.1038/s41378-022-00425-8 (PMC9424202; doi:10.1038/s41378-022-00425-8)
Supplement: Supplementary file 1 — Measuring nanoparticles in liquid with attograms resolution using microfabricated glass suspended microchannel resonator [file 41378_2022_425_MOESM1_ESM.pdf]

Supplementary notes

Measuring nanoparticles in liquid with attograms resolution using microfabricated glass suspended microchannel resonator

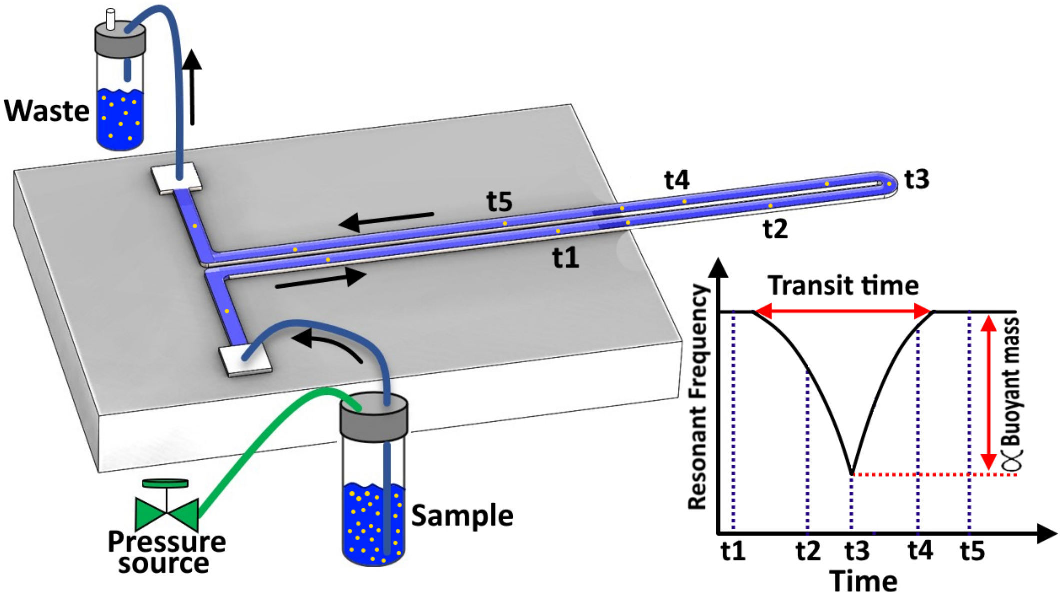

Mehdi Mollaie Daryani, Tomás Manzaneque, Jia Wei, Murali Krishna Ghatkesar

Department of Precision and Microsystems Engineering, Delft University of Technology, The Netherlands,
Department of Microelectronics, Delft University of Technology, The Netherlands

Contents

1 Device geometry **1**

2 Simulated and detected modes **2**

3 Detected modes and frequency spectra **3**

4 Setup **4**

5 SMR filling **5**

6 Au NPs detection **6**

1 Device geometry

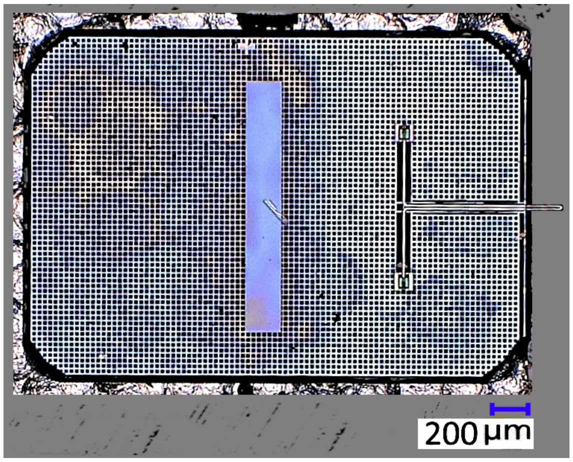

(a)

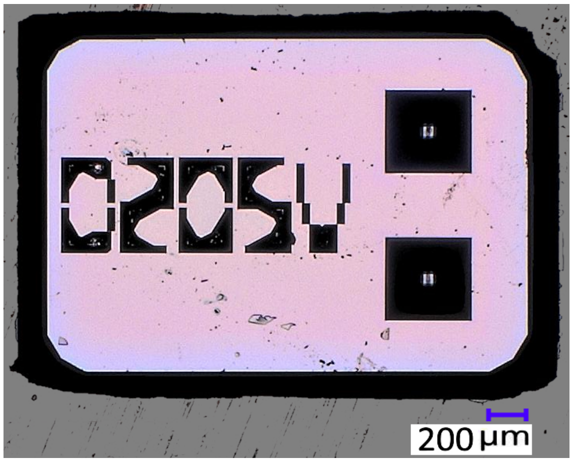

(b)

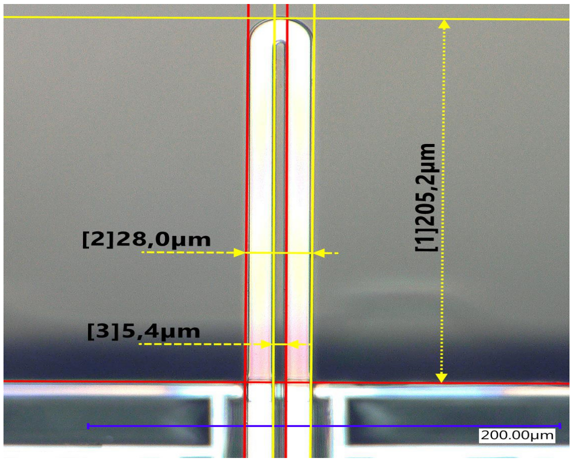

(c)

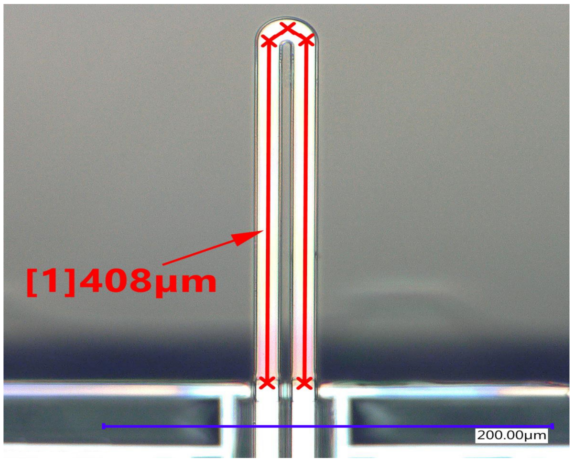

(d)

Figure 1: a) Top view of the chip and fluidic channels. b) Back side of the chip with two reservoirs shown in black squares, that connect the microfluidic channels to the world outside. c) and d) Top view of the U-shaped silicon dioxide resonator with dimensions.

2 Simulated and detected modes

This COMSOL study was performed based on a 3D model of our resonator with a Young's modulus of 87 GPa. The fact is that there are a range of values from 60-92 GPa for modulus of elasticity for SiO_2. Therefore, different modes irrespective of mode numbers would appear in very different resonance frequencies. We chose this value based on a study conducted on hollow microcantilevers at TU Delft (Belardinelli et al, 2017). Comparatively, the obtained eigenfrequencies show a discrepancy between simulation's (12 kHz, 674 kHz and 698 kHz) and the vibrometer's (149.42 kHz, 869.41 kHz and 893.29 kHz) for the first bending, first torsional and second bending modes respectively. We attributed this discrepancy to different values of Young's Modules and due to possible discrepancy in thickness of cantilever, which might be non-uniform through the cantilever.

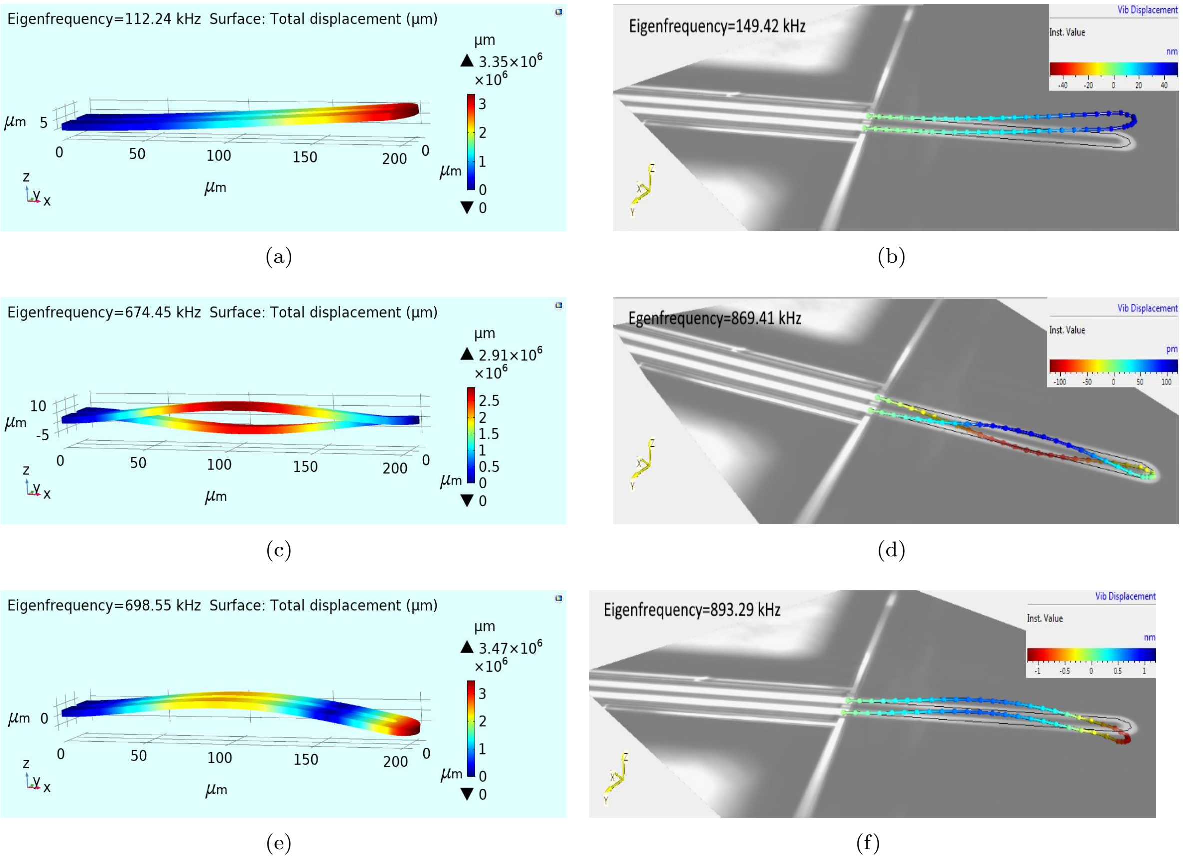

Figure 2: Results of eigenfrequency analysis by COMSOL (on the left) and their counterparts by the vibrometer (on the right) for a) and b) first bending mode, c) and d) first torsional mode, e) and f) second bending mode.

3 Detected modes and frequency spectra

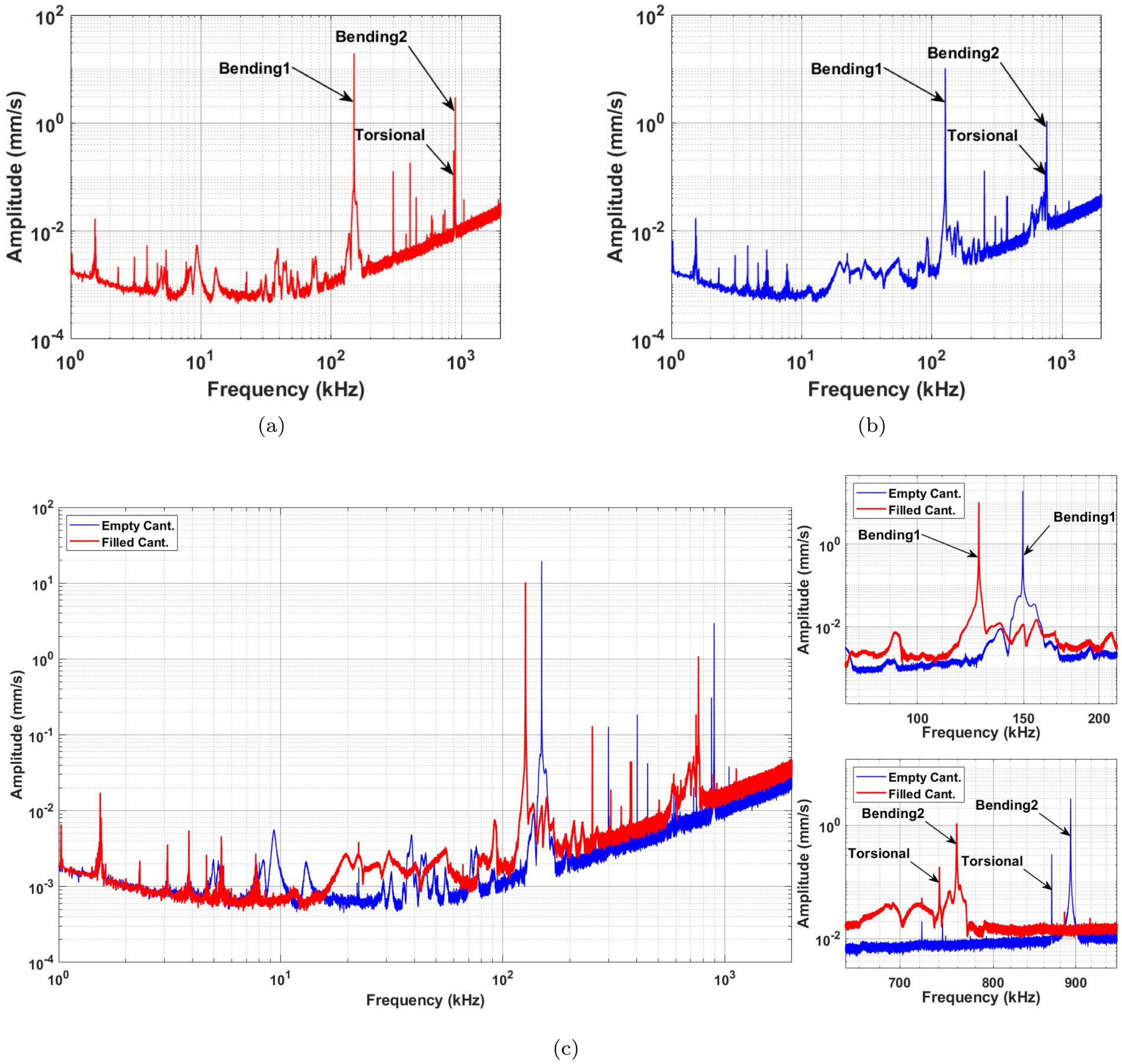

Figure 3: Complete frequency spectra, up to 2 MHz, of the a) empty, b) water-filled resonator and c) empty and water-filled cantilever together.

4 Setup

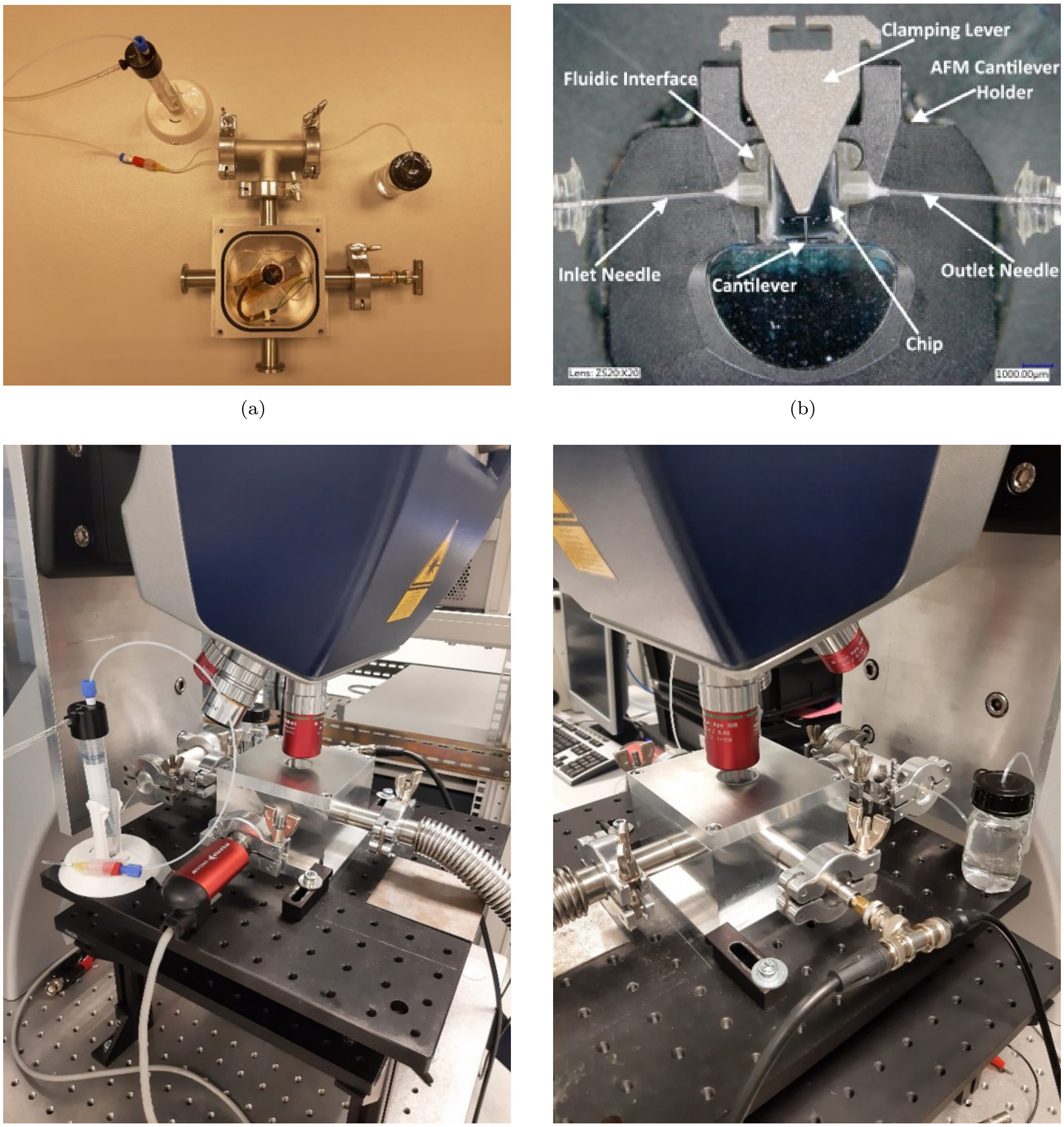

(a) (b) (c) (d)

Figure 4: a) The vacuum chamber (lid was removed) with its slots and connectors, b) a chip containing resonator, glued to an interface with needles, mounted on a Nanosurf AFM cantilever holder and placed inside the vacuum chamber. c) and d) show the setup on the workbench containing the vacuum chamber connected to fluids tubes, vacuum sensor, vacuum pump's hose and coaxial cables under a vibrometer objection.

5 SMR filling

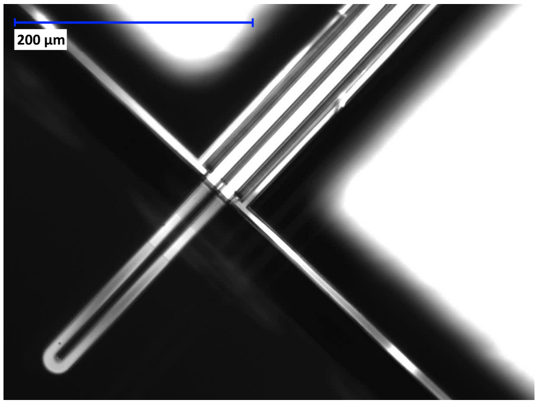

(a)

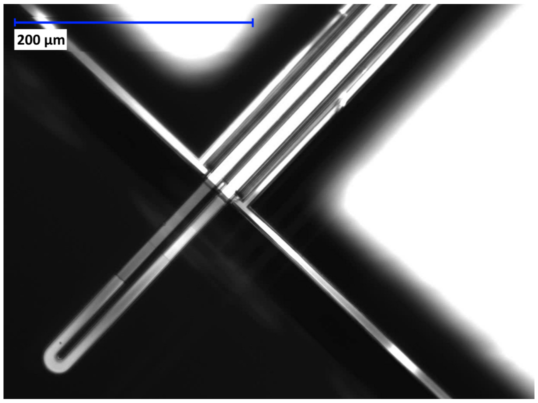

(b)

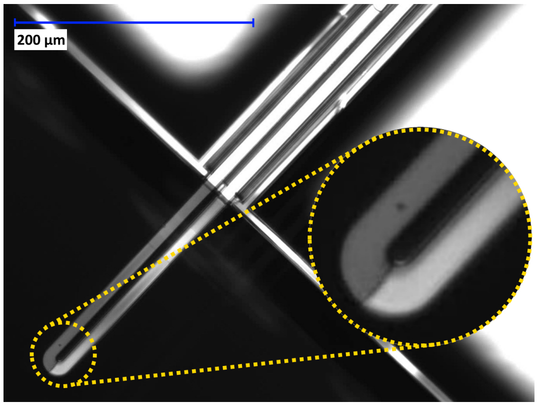

(c)

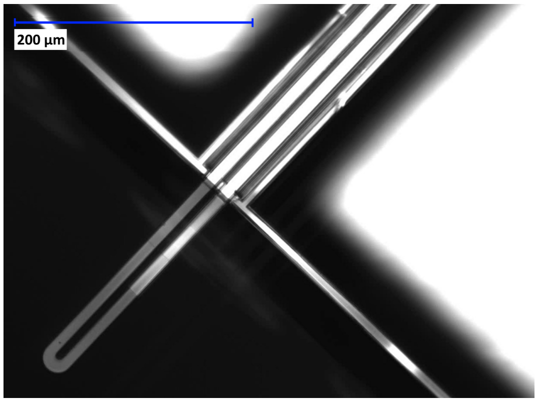

(d)

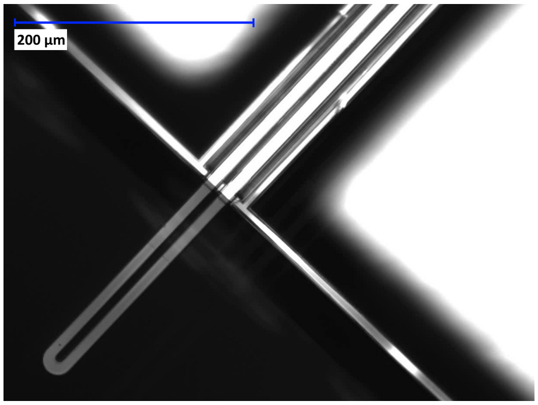

(e)

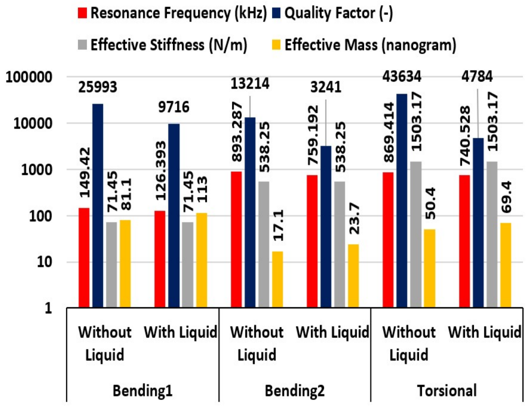

(f)

Figure 5: Suspended hollow cantilever being filled with liquid from left-leg to right-leg. The cantilever becomes dark when it is filled. The snapshots were taken when a) the cantilever was empty, b) the left leg was half-filled, c) the left leg was totally filled and the right leg was empty, d) the right leg was also filled in half and e) both legs were totally filled. f) Summary of the resonator's parameters for different modes, obtained from the modal characterization experiment.

6 Au NPs detection

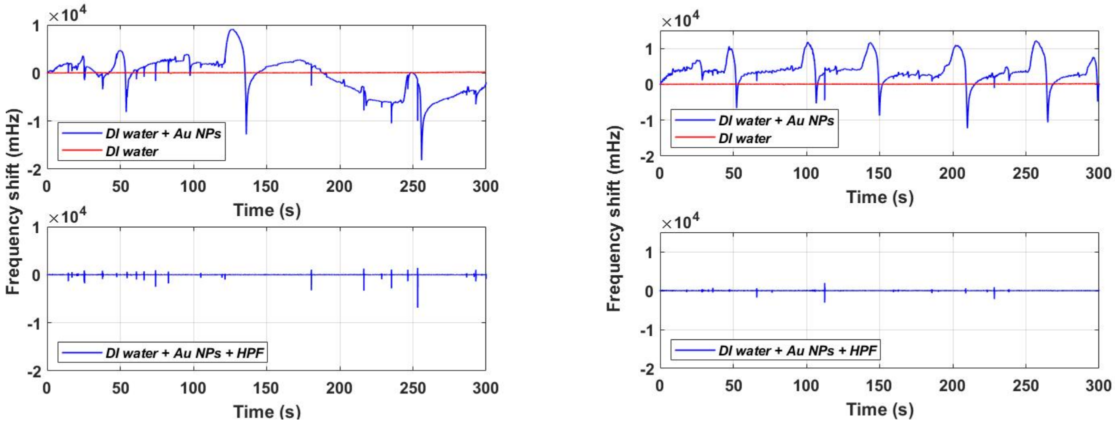

(a) (b)

Figure 6: Comparison of frequency shifts of two similar samples containing DI water and gold nanoparticles, while the driven pressure difference was a) 500 mbar and b) 1200 mbar. In both cases, small and tiny variations represent transmission of one or aggregates of Au NPs. It can be seen that large frequency variations are successively repeated in both cases, however the intensity is higher when the pressure difference is comparatively larger. These huge variations were removed by a high-pass filter (HPF), and were attributed to experiencing over pressure in the SMR.

References

Belardinelli P, Ghatkesar M, Staufer U, Alijani F (2017) Linear and non-linear vibrations of fluid-filled hollow microcantilevers interacting with small particles. International Journal of Non-Linear Mechanics 93:30 – 40, DOI https://doi.org/10.1016/j.ijnonlinmec.2017.04.016, URL `http://www.sciencedirect.com/science/article/pii/S002074621630364X`
